# Editorial: Women in performance science

**DOI:** 10.3389/fpsyg.2026.1764946

**Published:** 2026-02-04

**Authors:** Michiko Yoshie, Maria Varvarigou, Yuki Morijiri, Jennie Henley

**Affiliations:** 1 Human Informatics and Interaction Research Institute, National Institute of Advanced Industrial Science and Technology (AIST), Tsukuba, Japan; 2 Department of Human and Engineered Environmental Studies, Graduate School of Frontier Sciences, The University of Tokyo, Kashiwa, Japan; 3 Institute of Human Sciences, University of Tsukuba, Tsukuba, Japan; 4 Department of STEM Education, Mary Immaculate College, University of Limerick, Limerick, Ireland; 5 Graduate School of Teacher Education, Tokyo Gakugei University, Tokyo, Japan; 6 Royal Northern College of Music, Manchester, United Kingdom

**Keywords:** gender equality, music, pedagogy, performance science, psychology, wellbeing, women

We are delighted to present the Research Topic “*Women in Performance Science*” as a platform to promote the work of women scientists in the field of performance science. Women constitute only a minority of global researchers (UNESCO Institute for Statistics, 2020). This disparity stems from the persistent biases and gender stereotypes that dissuade women from pursuing science. Hence, this Research Topic has been launched to showcase women scientists' research across all sub-fields of performance science. Under this Research Topic, we have published 10 articles written by 31 authors from Australia, Canada, Japan, Portugal, the United Kingdom, and the United States. These articles include seven original research articles, two brief research reports, and a systematic review, encompassing diverse academic approaches, including pedagogy, psychology, physiology, and acoustics. A summary of each article is presented below.

Fujimoto and Uesaka examined how learning experiences can either promote or impede interpretive autonomy, and how this, in turn, influences the learning behaviors and wellbeing of eight elite piano and violin students. *Werktreue* internalization served as an analytical framework for the interview data, and its alignment with the self-determination theory emerged through the analysis. The findings suggested that interpretive autonomy fosters self-regulated learning and supports students' wellbeing. Furthermore, this study highlights that nurturing autonomy is essential from the earliest stages of music learning.

Goh et al. investigated gender marginalization within the Australian jazz and improvisation industries. Using a quantitative survey of 124 practitioners, they analyzed how symbolic boundaries reinforce male hegemony. Findings revealed that gender diverse individuals experience the most severe exclusion, while women report distinct challenges regarding work opportunities. Furthermore, awareness of sexual harassment was widespread across all genders. These results underscore the critical need for targeted inclusion strategies and enhanced safety measures to dismantle structural inequities.

Kondo et al. investigated the aspects of opera singing that influence overall performance evaluations by expert judges. They asked four experts to rate the performances of “Caro mio ben” by 10 trained female singers. Acoustic features such as singing power ratio and harmonic-to-noise ratio were also calculated. Consequently, the subjective rating of vibrato and the objective measurement of singing power ratio emerged as significant predictors of the overall performance score. This study contributes to elucidating the perceptual mechanisms underlying expert evaluations of opera singing.

Moura et al. reviewed solo music performance assessment systems, examining across instruments, rating methods, and target audiences. They highlighted the importance of the context, assessment purpose, and institutional culture, noting that both general and instrument-oriented methods can work effectively. Given that technique strongly shapes performance quality and expression, its weighting in assessment requires careful consideration. A shift from qualitative scales to detailed rubrics can improve the feedback for examiners and students. Finally, tailoring assessment tools for beginner, intermediate, and advanced students is important.

Perrier et al. examined the wellbeing of 16 professional female musicians using the self-determination theory. Participants reported mostly positive effects of being a musician, with autonomy and everyday experiences of mastery strongly supporting wellbeing. Lack of control and limited practice time created stress and reduced feelings of competence. Balancing work and life, especially decisions about having children, was a major challenge. Musicians developed resilience through self-compassion, organizational skills, and maintenance of focus. A deep love for music and transcendent performance experiences provided profound emotional and psychological satisfaction.

Sayers investigated pedagogical strategies for improvisation in North Indian classical music. Using audio-visual analyses of lessons in India, this study examined how oral transmission facilitates skill acquisition. Rather than being spontaneous, improvisation developed through the rigorous imitation and memorization of structured sequences, where “inexact replication” eventually fostered creative recomposition. These results underscore the cognitive sophistication of oral traditions, demonstrating that memory and implicit learning are fundamental for developing musical competence in non-notated systems.

Stephens-Himonides and Young investigated teacher identity within the Technological Pedagogical Content Knowledge (TPACK) framework. Through a case study of an experienced female music educator, they examined how personal background intersects with technology use in piano instruction. The study revealed that identity significantly shapes pedagogical choices and technology integration. Consequently, the authors argued in favor of extending the TPACK framework to include identity, providing a more holistic approach to supporting technology adoption in education.

Takagi et al. validated the Japanese version of the Kenny Music Performance Anxiety Inventory-Revised (K-MPAI-R). Data were collected from 400 Japanese musicians. An exploratory factor analysis extracted seven factors similar to those of the original scale. The scale demonstrated high internal consistency. Criterion-related validity was confirmed through strong correlations with other anxiety measures. Therefore, the Japanese version of the K-MPAI-R was considered a reliable and valid tool for research on music performance anxiety among Japanese musicians.

Watanabe et al. explored the optimal pre-performance state of professional musicians by examining the impact of memory recall. They asked 36 wind instrument players to recall positive or negative performance memories or imagine routine pre-performance activities. Recalling positive performance memories significantly enhanced subjective performance achievement, emotional arousal, and valence. The results suggested that positive recall fosters sympathetic activation and positive emotions, which collectively enhance performance achievement. This study provided objective evidence of the usefulneess of recalling positive performance experiences for professional musicians.

Yoshie and Morijiri investigated the impact of past and current social support on pre-performance mental states and performance quality among university music students. Students rated the support received from parents, a past teacher, and a current teacher, as well as their pre-performance self-confidence and performance evaluation for a recent major event. Past teacher support uniquely predicted self-confidence and performance quality, suggesting that the teacher support received by middle adolescence is particularly crucial for building student musicians' long-term self-confidence.

In conclusion, the articles within this Research Topic cover diverse topics in performance science, including musicians' wellbeing, pedagogical strategies, performance assessment, and gender equality. We believe that these studies, led by women scientists, will substantially contribute to advancing research in performance science.
